# 

**Published:** 1921-07-09

**Authors:** 


July 9, 1921] [Price Twopence

### The Hospital

The Workers' Newspaper of Administrative Medicine and Institutional Life, Administration, National Insurance and Health.

CONTENTS

**The King's Fund and a Hospital Policy** ... ... 239

**The Private Clinic System in Great Britain** ... 240
Sympathetic Encouragement to Group Clinics.

**Notes of the Week** 241
The Strength of Provincial Hospitals—Hospital Benefit and Approved Societies—Ireland and the Cave Commission—County Councils and Hospital Subscriptions—Free of Debt from its Foundation—A Sight for Hospital Visitors—The Right Spirit—The Prince and the Hospitals—Threat to Bradford Infirmary Site—A Chairman on Prices.

**Tonsils and Adenoids** ... ... ... ... ... ... 243

Some Light on a Recent Controversy.

**The Plea of Impulse** ... ... ... ... ... ... 244

**Bacteriological Pitfalls** ... ... ... ... ... 245

**Red Cross Conference on Venereal Diseases** ... 245

**Letters of a Layman** ... ... ... ... ... 246

Doctor and Patient.

**The Public Well-Being** ... ... ... ... ... 247

Maternity and Child-Welfare Centres.

**The British Hospitals Association** ... ... ... 249

Annual Meeting.

**The Inquiry System at Cardiff** ... ... ... 249

**Support through Local Committees** ... ... 250

**Parliament and the Professions** ... ... 250

**The Future of the Infirmary** ... ... ... ... 250

**A Successful Hospital Fete** ... ... ... ... 250

**Personalia** ... ... ... ... ... ... ... 250

**The Matrons' and Sisters' Department** ... ... 251

The Onus of Helping Necessitous Nurses.

**Round the Hospitals** ... ... ... ... ... 251

**Passing Events and Topics** ... ... ... ... 254

**Matrons' and Sisters' Appointments** ... ... vi

**The College of Nursing (Limited by Guarantee)** vi

**Forthcoming Events** ... ... ... ... ... ... vi

